# Metagenomics Reveals the Impact of Wastewater Treatment Plants on the Dispersal of Microorganisms and Genes in Aquatic Sediments

**DOI:** 10.1128/AEM.02168-17

**Published:** 2018-02-14

**Authors:** Binh T. T. Chu, Morgan L. Petrovich, Adit Chaudhary, Dorothy Wright, Brian Murphy, George Wells, Rachel Poretsky

**Affiliations:** a Department of Biological Sciences, University of Illinois at Chicago, Chicago, Illinois, USA; b Department of Civil and Environmental Engineering, Northwestern University, Evanston, Illinois, USA; c Department of Medicinal Chemistry & Pharmacognosy, University of Illinois at Chicago, Chicago, Illinois, USA; University of Tennessee and Oak Ridge National Laboratory

**Keywords:** antibiotic resistance, freshwater, lakes, metagenomics, wastewater treatment

## Abstract

Wastewater treatment plants (WWTPs) release treated effluent containing mobile genetic elements (MGEs), antibiotic resistance genes (ARGs), and microorganisms into the environment, yet little is known about their influence on nearby microbial communities and the retention of these factors in receiving water bodies. Our research aimed to characterize the genes and organisms from two different WWTPs that discharge into Lake Michigan, as well as from surrounding lake sediments to determine the dispersal and fate of these factors with respect to distance from the effluent outfall. Shotgun metagenomics coupled to distance-decay analyses showed a higher abundance of genes identical to those in WWTP effluent genes in sediments closer to outfall sites than in sediments farther away, indicating their possible WWTP origin. We also found genes attributed to organisms, such as those belonging to Helicobacteraceae, Legionellaceae, Moraxellaceae, and Neisseriaceae, in effluent from both WWTPs and decreasing in abundance in lake sediments with increased distance from WWTPs. Moreover, our results showed that the WWTPs likely influence the ARG composition in lake sediments close to the effluent discharge. Many of these ARGs were located on MGEs in both the effluent and sediment samples, indicating a relatively broad propensity for horizontal gene transfer (HGT). Our approach allowed us to specifically link genes to organisms and their genetic context, providing insight into WWTP impacts on natural microbial communities. Overall, our results suggest a substantial influence of wastewater effluent on gene content and microbial community structure in the sediments of receiving water bodies.

**IMPORTANCE** Wastewater treatment plants (WWTPs) release their effluent into aquatic environments. Although treated, effluent retains many genes and microorganisms that have the potential to influence the receiving water in ways that are poorly understood. Here, we tracked the genetic footprint, including genes specific to antibiotic resistance and mobile genetic elements and their associated organisms, from WWTPs to lake sediments. Our work is novel in that we used metagenomic data sets to comprehensively evaluate total gene content and the genetic and taxonomic context of specific genes in environmental samples putatively impacted by WWTP inputs. Based on two different WWTPs with different treatment processes, our findings point to an influence of WWTPs on the presence, abundance, and composition of these factors in the environment.

## INTRODUCTION

Wastewater is a source of antibiotic-resistant bacteria (ARB), antibiotic resistance genes (ARGs), and mobile genetic elements (MGEs) to the environment (1–4). ARB carrying ARGs have been isolated from lakes (5–7), rivers (8–10), and other aquatic environments (11, 12). Resistance proliferates in natural bacterial communities via horizontal gene transfer (HGT) (13–15) and can be transferred from aquatic environments back to human and animal microbial communities (16). Wastewater treatment processes aim to maximize the removal of nutrients, pathogens, and toxic compounds from wastewater before discharge into the environment. However, there is no regulatory standard and little monitoring for antibiotic concentrations, ARB, and ARGs in effluent, and wastewater effluent typically contains a variety of ARB, ARGs, and MGEs even after treatment (17–20).

Evidence for the impact of wastewater treatment effluent on the receiving aquatic environment has been investigated using culture-dependent methods that isolate ARB and PCR or quantitative PCR (qPCR) methods to assess the presence and abundance of specific ARB, ARGs, or MGEs (21, 22). For instance, using qPCR, LaPara et al. (23) found more tetracycline resistance genes, class 1 integrons, and Bacteroidetes in sites closer to a municipal wastewater treatment plant (WWTP) than in those farther away. Czekalski et al. (24) found up to a 200-fold higher abundance of some ARGs, as quantified by qPCR, in sites close to a WWTP than at remote reference sites. Devarajan et al. (6) also observed a strong correlation between the abundance of fecal indicator bacteria and ARGs in Lake Geneva (central Europe) with organic matter and metal concentration in an area close to a WWTP. A limitation of using culture-dependent methods is that only a small portion of ARB can be isolated (25). In addition, cultivation-independent PCR-based methods require the use of dedicated primers and thus allow the detection or quantification of only certain targeted ARGs from a sample. To overcome the limitations and biases of cultivation- and PCR-based methods, shotgun metagenomic analysis offers a PCR-independent methodology that can be used to gain a broader understanding about the presence and abundance of many different genes and organisms within a sample. In one recent study that used a clone-based metagenomics approach in a WWTP and effluent-receiving waterway, more antibiotic resistance clones were recovered closer to the WWTP than farther downstream (10). Metagenomics has also been used to show a higher abundance of potential pathogens and ARGs in river water near an effluent outfall than elsewhere in the river (26) and a high abundance of ARGs belonging to aminoglycoside and phenicol groups, which include chloramphenicol and its derivatives florfenicol and thiamphenicol, in river water that received WWTP effluent (27).

Although recent metagenomic approaches have provided evidence for the impact of WWTP on effluent-receiving water bodies, none have simultaneously tracked microorganisms, ARGs, and other genes from a WWTP into the environment by linking organisms and genes. Here, we present a comprehensive analysis of the abundances of ARGs, MGEs, and microbial communities, including putative pathogenic microorganisms and their virulence factors, in the effluent of two full-scale WWTPs that use two different treatment processes (trickling filter and activated sludge). Furthermore, we examine the dispersal of these factors in Lake Michigan, the recipient of effluent discharge from these plants. Our results support previous reports of possible antibiotic gene dissemination from WWTPs to the environment and expand our knowledge about the overall influence of WWTPs on both gene content and microbial community composition in a receiving water body. Furthermore, our characterization of ARG-carrying taxa and ARGs associated with plasmids provides evidence for a mechanism of dispersal and proliferation of WWTP genetic elements in the environment.

## RESULTS

### WWTP effluent influences the overall genetic composition of lake sediments.

Before looking for evidence of specific genes released from WWTPs to Lake Michigan, we assessed the impact of WWTP effluent on the overall genetic profile of sediment samples in the lake. We directly mapped the sediment metagenomes against effluent metagenomes by BLASTP searching all amino acid sequences from assembled genes from the sediments collected around WWTPs against those of WWTP effluents. We searched for identical sequences (100% identity of 100% alignment length) between sediments and effluent, presuming that this stringent threshold provides a conservative estimate of genes that likely originate from WWTPs. From this, we estimate that 0.002 to 0.013% of the tens of thousands of genes in Manitowoc sediment metagenomes (Table S4) and 0.05 to 0.09% of genes in Sheboygan sediment metagenomes originated from the WWTPs.

**FIG 1:**
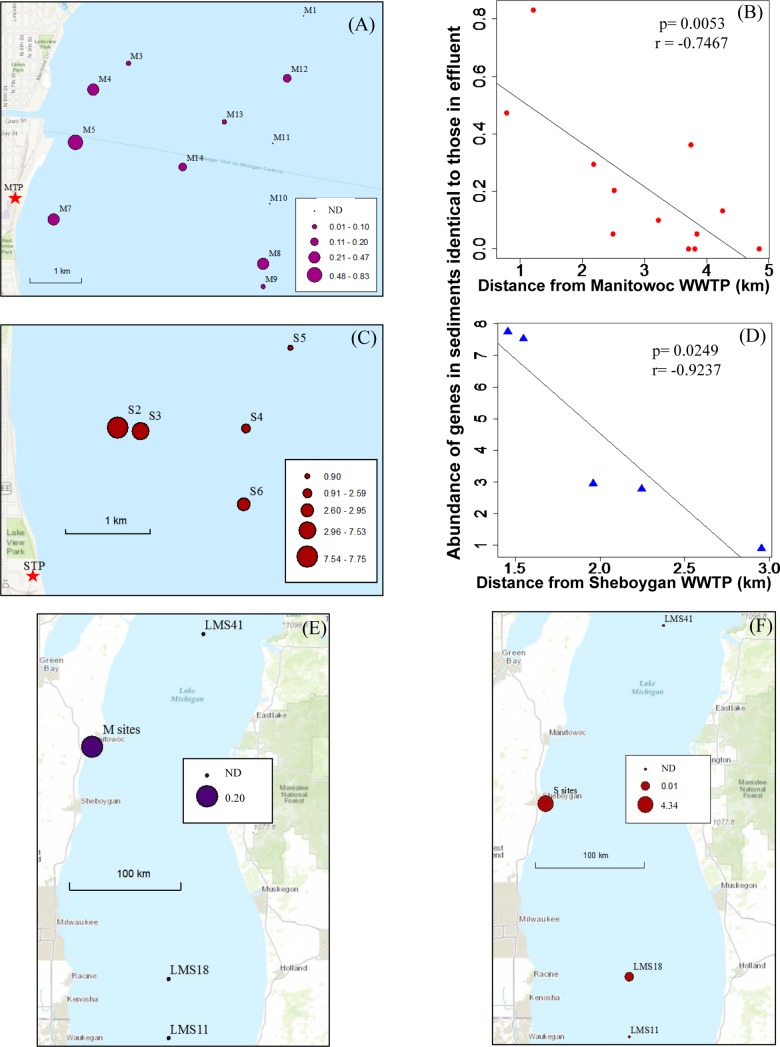
Normalized abundance of genes found in Lake Michigan that are identical to those from WWTP effluent from Manitowoc (A) and Sheboygan (C), and the correlation between identical gene abundance with the distance from WWTPs (B and D). (E and F) Mean abundances of those genes from Manitowoc sites E) (all sites from panel A are combined as “M sites”) and from Sheboygan sites (F) (all sites from panel C are combined as “S sites”) are shown along with abundances from reference samples (LMS11, LMS18, and LMS41) for comparison. ND, not detected. Filled circles show the scale of gene abundance. Red stars mark the positions of the Manitowoc WWTP (MTP) and Sheboygan WWTP (STP). (Source: Esri.)

For both Manitowoc and Sheboygan, a higher proportion of identical genes were observed at sites closer to the WWTPs than those that were farther away, with a significant negative correlation between the proportion of identical genes in sediments and their distance from effluent outfall (*P* = 7.6e−6, *r* = −0.936 for Manitowoc and *P* = 0.00015, *r* = −0.997 for Sheboygan sediments; Fig. S2). In addition to contributing to a greater proportion of the metagenomes closer to the WWTPs, identical genes were also more abundant in these samples. Sediment from sites M5 and M7, which were closest to the Manitowoc WWTP, had the highest abundance of genes identical to those from effluent (Fig. 1A and B). No identical genes were found in sediment samples from sites M1, M10, and M11, which were farther from the WWTP. Although we collected fewer Lake Michigan sediment samples in close proximity to the Sheboygan treatment plant, we observed a similar trend of higher abundance of identical genes at sites located closer to the WWTP (Fig. 1C and D). Overall, the abundance of identical genes from sediment samples was significantly correlated with distance from WWTPs (correlation coefficients, *r* = −0.747 and −0.924 for the Manitowoc and Sheboygan sites, respectively; *P* = 0.0053 for Manitowoc and *P* = 0.0249 for Sheboygan; Fig. 1B and D). To examine whether these genes were present in the environment with limited or no WWTP influence, we included three reference samples that were collected in the middle of Lake Michigan. There were few to no identical genes found in these samples, which were obtained 100 to 200 km from the WWTPs (Fig. 1E and F). This suggests that based on the composition of the effluent at the time of our sampling, there was minimal influence of WWTP effluent on sites that were distant from effluent outfalls.

The putative taxonomic origin of these identical genes was determined. All of the identical genes between Manitowoc effluent and sediments were attributed to bacterial genomes, while in Sheboygan samples, some (18 to 36%, depending on the sample) could either not be classified using the genome reference database or were not assigned to bacteria (data not shown). Most of the identical genes across both WWTPs and their neighboring sediment samples were attributed to unclassified proteobacteria or to Proteobacteria belonging to Pseudomonas, Xanthomonas, and Acidovorax; 100% of the identical genes in eight Manitowoc sediment samples were proteobacteria. In addition, 16 to 33% of the identical genes between the Sheboygan WWTP and sediments were identified as cyanobacteria. No identical ARGs were identified between WWTP effluent and sediments.

### Impact of WWTP effluent on the bacterial composition and abundance in Lake Michigan sediment.

We next compared bacterial community structure, inferred from genetic composition, between WWTP effluents and the corresponding sediment samples. Approximately half of the genes (ranging from 39 to 68%; 32 to 87% when normalized for abundance) identified in each metagenome could be assigned to a taxon at the genus level, with the highest proportions being in sediment samples taken from the middle of Lake Michigan (Fig. S4). At the phylum level, Proteobacteria were dominant in all samples, followed by Bacteroidetes and Firmicutes in wastewater or by Cyanobacteria, Bacteroidetes, Nitrospirae, Firmicutes, and Actinobacteria in sediment samples (Fig. S3). Although WWTP effluent does not appear to influence the overall sediment bacterial composition and abundance at the phylum level, we identified specific phyla that were abundant in WWTP effluent and significantly decreased in relative abundance (normalized to mean coverage of 108 single-copy genes) in sediments with increased distance from effluent outfalls. These included Bacteroidetes, Fusobacteria, and Spirochaetes in Manitowoc and Chlamydiae and Firmicutes in Sheboygan (Table 1). Several phyla showed a significantly higher relative abundance with increasing distance from effluent outfalls, including Cyanobacteria, Chrysiogenetes, Nitrospirae, and Nitrospinae in Manitowoc and Proteobacteria in Sheboygan (Table 1). Because metagenomic data are inherently compositional, it is impossible to conclude from these relative abundances alone whether the numbers of these groups of organisms actually increased or decreased with increasing distance from the WWTP; however, the overall diversity of each sediment sample was generally similar between the Manitowoc and Sheboygan sites, respectively (see Fig. S1 in the supplemental material), indicating that the observed changes in relative abundances are meaningful.

**TABLE 1:** Pearson correlation coefficients between normalized abundance of phyla and the distance from Manitowoc and Sheboygan effluent outfalls^*a*^

Phylum	Manitowoc		Sheboygan	
	*r*	*P* value	*r*	*P* value
Bacteroidetes	−0.588	0.034		
Fusobacteria	−0.607	0.028		
Spirochaetes	−0.554	0.049		
Cyanobacteria	0.599	0.031	0.797	0.057
Chrysiogenetes	0.856	0.0002		
Nitrospinae	0.676	0.011		
Nitrospirae	0.832	0.0004		
Chlamydiae			−0.871	0.024
Firmicutes			−0.798	0.057
Proteobacteria			0.908	0.012

a Significant (*P* < 0.05) correlations are shown, along with correlations that are nearly significant. The correlation coefficient (*r*) of <0 shows the decreased abundance with the increased distance from WWTP.

Proteobacteria and Bacteroidetes were the most abundant phyla in both WWTP effluents and sediments. We therefore looked for families and genera within these phyla that showed significant negative correlations between abundance and distance from WWTPs. We postulate this to reflect the impact of WWTP effluent on Lake Michigan, as it represents bacterial taxa that were abundant in WWTP effluents but decreased in sediments farther from the corresponding WWTPs. Within the Bacteroidetes, we observed two genera, Bacteroides and Flavobacterium, that were the most abundant in Manitowoc WWTP effluent and decreased in sediments with increased distance from treatment plant. Some families and genera, such as Helicobacteraceae (Helicobacter), Legionellaceae (Legionella), Moraxellaceae (Acinetobacter and Moraxella), and Neisseriaceae (Neisseria) were abundant in both Manitowoc and Sheboygan WWTP effluent and showed a significant negative correlation between abundance and distance from WWTP effluent outfalls (Table 2). Other families and genera displaying these significant negative correlations were specific to WWTP effluent: Aeromonadaceae (Aeromonas), Campylobacteraceae, and Enterobacteriaceae (Escherichia and Yersinia) from Manitowoc and Francisellaceae (Francisella), Methylophilaceae, and Rickettsiaceae (Rickettsia) from Sheboygan (Table 2).

**TABLE 2:** Proteobacterial families and genera found in WWTP effluents and sediments showing decreased abundance with increased distance from WWTPs

Taxon	Manitowoc		Sheboygan	
	*r*	*P* value	*r*	*P* value
Aeromonadaceae	−0.791	0.0013		
Aeromonas	−0.787	0.0014		
Campylobacteraceae	−0.569	0.0422		
Enterobacteriaceae	−0.755	0.0028		
Escherichia	−0.707	0.0068		
Yersinia	−0.734	0.0043		
Helicobacteraceae	−0.563	0.0450	−0.867	0.0255
Helicobacter	−0.595	0.0319	−0.870	0.0243
Legionellaceae	−0.757	0.0027	−0.933	0.0066
Legionella	−0.747	0.0033	−0.934	0.0064
Moraxellaceae	−0.684	0.0099	−0.968	0.0016
Acinetobacter	−0.6795	0.0106	−0.913	0.0110
Moraxella	−0.6319	0.0205	−0.957	0.0027
Neisseriaceae	−0.734	0.0043	−0.934	0.0065
Neisseria	−0.6358	0.0195	−0.935	0.0062
Francisellaceae			−0.938	0.0056
Francisella			−0.938	0.0056
Methylophilaceae			−0.969	0.0014
Rickettsiaceae			−0.932	0.0068
Rickettsia			−0.932	0.0067

Because many of the sediment families and genera that appeared to originate from the WWTPs have pathogenic relatives (28), we looked for the presence of genes encoding bacterial virulence factors from these organisms in both effluent and environmental samples. Cross-referencing taxonomic annotations from MyTaxa and the Virulence Factor Database (see Materials and Methods), we determined that virulence factor-like genes from Manitowoc WWTP effluent were most closely related to those from Aeromonas, Acinetobacter, Legionella, and Pseudomonas spp., while in Sheboygan WWTP effluent, they were associated with more diverse bacterial taxa within the genera Acinetobacter, Burkholderia, Legionella, Mycobacterium, Pseudomonas, and Rickettsia. A variety of virulence factor-like genes were identified; however, most of the ones we observed encode a range of products, not all of which are exclusively pathogenic (e.g., those related to host association). While we are therefore unable to conclusively determine whether or not these organisms are pathogenic, queries of virulence factor-like gene distributions provided further evidence of WWTP influence on overall gene content; in 7 of 12 Manitowoc sediments, we found sequences with >90% identity along >90% alignment length to Manitowoc WWTP effluent virulence factor-like genes, and 4 of 5 Sheboygan sediment samples contained virulence factor-like sequences with >80% identity along >90% alignment length to those in effluent; however, no virulence factor-like genes matching those in effluent samples were found in the middle of the lake at these same stringent cutoffs.

### Influence of WWTP effluent on the composition and abundance of antibiotic resistance genes.

None of the ARGs observed in sediment metagenomes matched effluent ARGs identically (i.e., 100% identity over 100% sequence alignment length), likely due to the highly microbially diverse populations and relative low ARG abundances in sediments (Fig. S1). Therefore, in order to compare ARG profiles, we instead tracked ARGs from effluent to lake sediments by the class of antibiotic to which they contribute resistance, as well as at the level of individual ARGs using a less stringent threshold (E value threshold of e^−10^, 70% sequence similarity cutoff, and bit score of 50) based on a combination of previously accepted cutoffs for tracking ARGs in the environment as well as our own comparisons of individual genes using digital droplet PCR (ddPCR) and in silico analyses (see Materials and Methods and the supplemental material). While these thresholds might capture ARGs that originated from the WWTPs in addition to those that were similar to effluent ARGs, they were sufficient for identifying ARGs and exploring overall similarities and differences between locations.

**FIG 2:**
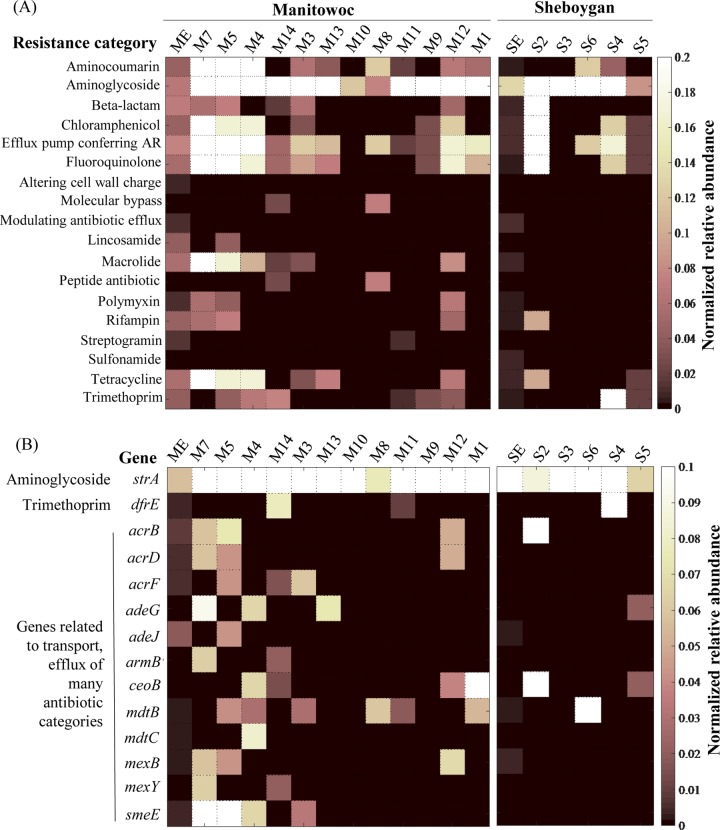
Normalized relative abundance (to mean coverage of 108 single-copy genes) of ARG categories in WWTP effluent and sediments (A) and of shared specific ARGs that appear in at least one sediment sample and effluent (B). ME and SE, effluent from Manitowoc and Sheboygan WWTPs, respectively; M1 to M14, Lake Michigan sites around Manitowoc WWTP; S2 to S6, sites around Sheboygan WWTP. The order of the sites from left to right corresponds to increasing distance from WWTPs. Locations of the sampling sites can be found in Fig. 1A and C.

Sixteen of the 28 ARG categories in the Comprehensive Antibiotic Resistance Database (CARD) were detected in the effluent from both WWTPs (Fig. 2A). The effluent ARG profiles for the two WWTPs were similar, but Manitowoc WWTP effluent ARGs were generally twice as abundant as those in the Sheboygan WWTP. Sediment samples collected closer to the Manitowoc WWTP (sites M5 and M7) and the Sheboygan WWTP (site S2) exhibited higher ARG diversity and abundances than did samples from sites located farther from the plants (Fig. 2). Overall, 41 distinct ARGs were detected, accounting for 0.012% of the Manitowoc WWTP effluent metagenome, while 17 (0.013%) ARGs were detected in Sheboygan WWTP effluent. Except for *dfrE* (specific for trimethoprim resistance) and *strA* (specific for aminoglycoside resistance), most of the identified ARGs encode multiresistance efflux pumps (Fig. 2B). For example, the *acrB*, *adeJ*, *mexB*, and *smeE* genes encode proteins that transport more than six categories of antibiotics each. Among ARG categories, aminoglycoside resistance genes were the most abundant in effluents from both WWTPs and in all samples but the Manitowoc M8 sediment samples. Other ARG categories, such as efflux pumps conferring resistance and fluoroquinolone, chloramphenicol, tetracycline, trimethoprim, and macrolide resistance, were also abundant in WWTP samples and detected in between 6 and 15 of the 17 sediment samples (Fig. 2A). Most effluent ARGs (56 to 82%) were attributed to Proteobacteria, followed by Firmicutes (5.5 to 12%) and Bacteroidetes (3 to 10%) (Fig. S4).

Several ARGs were more abundant in sediment samples than in the WWTP effluents (Fig. 2). These included an aminoglycoside resistance gene, *strA*, which was more abundant in all 12 Manitowoc sediment samples and in three of the five Sheboygan sediments than in WWTP effluents (Fig. 2B). Other ARGs related to resistance to aminocoumarin, chloramphenicol, fluoroquinolones, macrolides, or tetracycline or ARGs encoding efflux pumps were more abundant in sediments collected from sites M4, M5, and M7, which are closer to the Manitowoc WWTP, than the other sediment samples (Fig. 2A). Genes encoding efflux pumps and resistance to beta-lactams, chloramphenicol, fluoroquinolones, rifampin, and tetracycline were also more abundant in the sediment sites closer to the Sheboygan WWTP.

**FIG 3:**
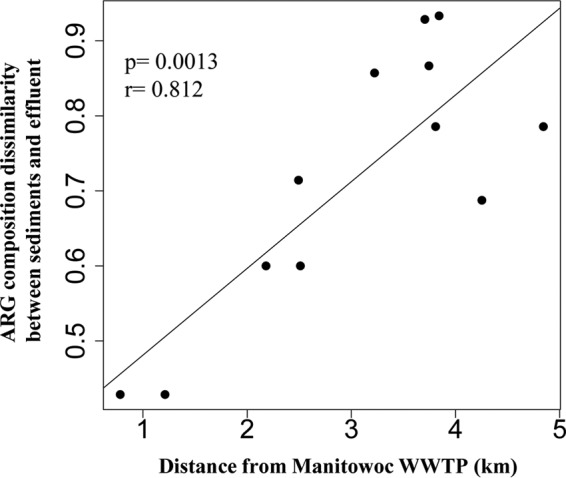
Correlation between geographic distance and Jaccard dissimilarity index for shared ARG composition in sediments and Manitowoc WWTP effluent.

Distance-decay analysis was used to assess the impact of WWTPs on the composition and abundance of ARGs in lake sediments. While a distance-decay relationship was not observed for ARG abundance, we found a significant correlation (*P* = 0.0013; correlation coefficient, *r* = 0.812, Jaccard dissimilarity) between the distance of lake sediments from Manitowoc WWTP and ARG composition. Specifically, the farther from the WWTP, the more dissimilar the sediment ARG composition (Fig. 3). This indicates a plausible influence of the Manitowoc WWTP effluent on the ARG composition in surrounding sites in Lake Michigan, either through the introduction of genes or alteration of the chemical environment. Similar analyses were done for the Sheboygan sites, but we did not observe a significant correlation between ARG composition or abundance and distance from the Sheboygan WWTP (*P* = 0.45 and 0.66, respectively). This might be due to the higher relative abundance of ARGs in sediment (Fig. 2) than in effluent, together with the limited number of sediment samples that we obtained for Sheboygan sites.

**FIG 4:**
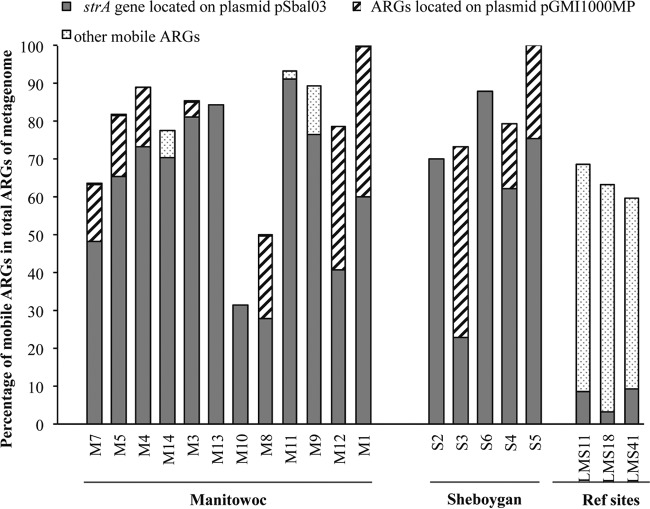
Percentage of mobile ARGs that are located on plasmids in total ARGs detected from sediments near the WWTP effluent outfall (sites M1 to M14 and S2 to S6) and reference sediment samples (LMS11, LMS18, and LMS41). The order of the sites from left to right corresponds to increasing distance from WWTPs.

ARGs are easily transferred among organisms of different taxa via HGT if they are located on plasmids (29). To determine which ARGs were located on plasmids, we mapped blast results from the ACLAME plasmid database with blast results from the CARD for each metagenomic data set. Genes found in both blast results were considered ARGs located on MGEs. High proportions of ARGs were found on plasmids in Manitowoc and Sheboygan WWTP effluent samples (M. Petrovich, B. T. T. Chu, D. Wright, J. Griffin, M. Elfeki, B. T. Murphy, R. Poretsky, and G. Wells, unpublished data); here, we also observed high proportions of plasmid-associated ARGs in sediments, ranging from 32% (M10) to 100% (M1 and S5) of the identified ARGs (Fig. 4). The *strA* gene encoding aminoglycoside resistance and located on plasmid pSbal03 was the most abundant mobile ARG in effluents of both WWTPs and in all sediment samples collected surrounding the WWTPs. It was also the only mobile ARG found in some sediments (M10, M13, S2, and S6). We found some *strA* sequences associated with pSba103 in samples collected from the middle of the lake; however, their proportion in the total ARGs is much higher in the sites surrounding WWTPs than in the middle of the lake (Fig. 4). This may reflect the influence of the WWTPs on the dispersal of this MGE-associated ARG, although we cannot exclude the possibility that it might also originate from other sources near shore. Another plasmid, pGMI1000MP, which carries *acrB*, *mdtB*, *mdtC*, *ceoB*, *mexD*, *mexY*, and *smeE*, all of which encode multidrug resistance efflux proteins, was also found in WWTP effluents and in 10 out of 18 sediment samples. No ARGs on plasmid pGMI1000MP were found in reference samples that were collected in the middle of Lake Michigan. Although the ecological importance of the MGEs pSbal03 and pGMI1000MP has not been studied, their abundance in effluent samples and their ubiquity in sediment samples may indicate that they are persistent through the treatment process and become a vehicle for spreading ARGs into the environment.

We further queried which microorganisms carry mobile ARGs, and thus whether these organisms might be responsible for transferring ARGs to sediment organisms via HGT. Surprisingly, there was a high proportion of mobile-ARG-conferring taxa within ARG-carrying populations in sediment samples; almost all ARG-carrying bacteria in samples M1, M11, and S5 confer mobile ARGs (Fig. S5). Proteobacteria were commonly found in mobile-ARG-carrying populations and were primarily affiliated with the genera Burkholderia and Pseudomonas in both WWTP effluent and sediments. Cyanobacteria were also commonly found to confer mobile ARGs in sediment samples. The *strA* gene, encoding aminoglycoside resistance, which was the most abundant mobile ARG found in effluents and sediments, was carried by bacteria of different taxa, not all of which were the same between the effluent and sediment samples. For example, in Manitowoc WWTP effluent, it was carried by different Proteobacteria, Bacteroidetes, and Actinobacteria, whereas in most Manitowoc sediments, it was found in Cyanobacteria and Proteobacteria but not Actinobacteria.

## DISCUSSION

Using metagenomic sequencing of WWTP effluent and sediment samples, we showed a significant negative correlation between the abundance of identical genes from sites around WWTPs and their distance from effluent outfall, indicating that WWTP effluent exerts an influence on sediment genetic composition. This likely includes the antibiotic resistance genes and mobile genetic elements that are present in WWTP effluent, although the low relative abundances of these genes in the sediment along with their sequence divergence made it difficult to unequivocally link them to the WWTP. Nevertheless, we identified similar patterns of dispersal and WWTP influence from two different WWTPs that employ different secondary treatment processes: trickling filter- and activated-sludge-based treatment. Although the Manitowoc effluent metagenome had more ARGs both within and between ARG classifications than did the Sheboygan effluent, we observed similar effects of the two WWTPs on the surrounding environment. Regardless of the composition of each respective effluent, the sediment metagenomes as a whole were reflective of the WWTP to which they were closer. Our results corroborate previous studies showing a higher abundance of specific ARGs in sites around WWTPs than in sediment or water from sites farther away (10, 26, 27), and they significantly extend our knowledge of WWTPs regarding gene content and organisms beyond just ARGs.

Although we observed distinct patterns between genes that were common to sediment and effluent, we were not able to identify any of these identical genes as ARGs. This might be because our blast threshold of 100% identity over 100% sequence alignment length is too stringent, or, likely, that the number of ARGs in the sediment was below our detection limit. The sediment samples had high diversity and substantially lower coverage than the WWTP samples (Fig. S1), so our chances of capturing identical ARGs in the sediment samples were low. For instance, based on digital droplet PCR (ddPCR, described in the supplemental material) quantification of several ARGs in WWTP effluents and lake sediments, we would have needed ∼10-Gb sequencing depth to detect a single copy of *sul1* (a sulfonamide resistance gene) in effluent and sequencing depth at hundreds or even thousands of Gb to detect one copy of this gene in sediments (Table S2). Nevertheless, we were able to follow the influence of WWTP effluent on ARGs in sediment samples by examining genes assigned to ARGs using standard (nonidentical) cutoffs. Among these were the high proportion of ARGs carried by MGEs in both effluent (>40% of total ARGs) and sediments (>30% of total ARGs), indicating a propensity for ARG mobility and the possible influence of WWTPs on these genes in the environment. Using qPCR and focusing on a subset of just six ARGs encoding sulfonamide and tetracycline resistance, Czekalski et al. (24) observed a distance-decay relationship between a WWTP and sites in Lake Geneva. Here, we expanded on these findings by comparing all annotated ARGs and finding a significant distance-decay relationship between the effluent outfall of Manitowoc WWTP and the composition of the ARGs.

In addition to finding that there were more ARGs in sites closest to the treatment plants than in those farther away, we observed that several of these genes were more abundant in these sediments than in the effluent. Similar observations were made in a different system where the abundances of aminoglycoside and phenicol resistance genes were higher in the environment that received WWTP effluent than that in the effluent itself (27). The higher abundance of ARGs in sediments might be a result of (i) bioaccumulation of ARGs over time, (ii) proliferation in the sediment due to multiplication of organisms carrying ARGs that survived the treatment process and/or spread of genes via HGT, (iii) selective pressure in the sediment through competition and/or signaling to increase naturally occurring ARGs, or (iv) introduction of ARGs from other sources besides the WWTPs. For instance, the Manitowoc River (∼1 km north of the Manitowoc WWTP) and the Sheboygan and Black Rivers (∼5 km north and ∼2 km south of the Sheboygan WWTP, respectively) flow into Lake Michigan and likely introduce ARGs to the lake.

The actual number of genes and organisms of concern in WWTP effluent was found to be low; however, WWTPs can still have a profound impact on the environment. Quantification of a single-copy gene (*rpoB*) by ddPCR showed that there are >10^6^ bacterial cells found in 1 liter of each WWTP effluent (Table S3). The average capacity of the Manitowoc WWTP is 9 million gallons per day (MGD) (http://www.manitowoc.org/511), and that of the Sheboygan WWTP is 10 MGD (http://www.sheboyganwwtp.com/9b_Data.php), equivalent to 34 to 37.8 million liters per day. Therefore, each of these two WWTPs release ∼34 × 10^12^ to 37.8 × 10^12^ bacterial cells (viable or nonviable) to Lake Michigan per day. Our data suggest that among the genes introduced to sediments from organisms present in WWTP effluent are some virulence factor-like genes; however, it is difficult to determine whether these are (i) viable and (ii) disease causing. Because taxonomic identification of gene sequences depends on existing databases that are skewed toward pathogens, and because the Virulence Factor Database includes only pathogens, organisms might be close relatives of known pathogens without being pathogenic themselves. Furthermore, many virulence factors are involved in processes that are not exclusively pathogenic, such as cell-host interaction and surface adhesion. Deeper metagenomic coverage would be needed to better compare taxa between samples and existing complete and draft genomes.

Although none of the ARGs we identified in the sediment samples were 100% identical to those in effluent for reasons discussed above, the patterns of ARGs and gene classes suggest a likely influence of WWTP effluent on the composition ARGs that are found in the immediate vicinity. Metagenomic data alone are not sufficient to determine whether these patterns are due to complex microbial interactions in the environment, shifting physicochemical conditions, or solely the introduction of specific genes and organisms, but we suspect that a combination of these factors is important. Based on our estimation that ∼30 × 10^12^ bacterial cells per day are released into Lake Michigan at each of the WWTP discharge sites studied here (see above), we calculated that 0.016% carry ARGs, 46 to 76% of which are located on MGEs. This equates to ∼5 × 10^9^ to 6 × 10^9^ ARG-carrying cells from WWTP effluent released into Lake Michigan every day. Therefore, although the relative abundance of ARGs in effluent is low, they are amplified by the 9 to 10 MGD of wastewater effluent discharged into the lake. The transfer frequency of ARGs via MGEs within environmental bacterial communities is largely unknown, but we found a high proportion (ranging from 32 to 100% of all ARGs) of bacteria carrying mobile ARGs in sediment samples (Fig. S5). The existence of these ARGs on MGEs might be especially important in the spread and accumulation of ARGs once they reach the lake.

Surprisingly, many sediment ARGs were affiliated with Cyanobacteria. Although Cyanobacteria are ubiquitous in aquatic environments (30), antibiotic resistance in Cyanobacteria in the environment has not been studied as much as antibiotic resistance in other phyla, such as Proteobacteria, Firmicutes, and Bacteroidetes (31–33). Some cyanobacterial strains have shown antibiotic resistance activity *in vitro* (34, 35). Our metagenomic analysis confirmed the antibiotic resistance potential of Cyanobacteria in aquatic systems, although we were not able to get detailed information about which cyanobacterial genera or species possess ARGs, as we were only able to classify genes to the phylum level. It might be possible for ARGs introduced into the environment via effluent to be transmitted to environmental cyanobacteria, particularly given the high proportion of observed ARGs associated with MGEs.

Our results demonstrate the probable influence of WWTPs on the composition of genes and organisms in sediments near WWTP outfalls and provide important insight into the bacterial communities, especially those that harbor ARGs and MGEs, in wastewater effluent and receiving water bodies. Using a metagenomics approach, we were able to link taxa and their genes within complex communities and establish their relationship with the location in which they were found. Given the overall similarity between WWTP effluent gene content and that of sediments closer to effluent outfalls, as well as the abundance and diversity of ARGs and MGEs within the WWTP effluent microbial community, we propose that bacteria originating in WWTP effluent have the potential to disseminate genes, including antibiotic resistance genes, via HGT in the environment.

## MATERIALS AND METHODS

### Sample collection.

Effluent samples were collected in July 2015 from two WWTPs in Wisconsin, USA: the Manitowoc WWTP (44.0836454°N, −87.6555826°W) and the Sheboygan WWTP (43.718756°N, −87.710559°W). These two WWTPs use different secondary treatment processes: Manitowoc WWTP uses a trickling filter, while Sheboygan WWTP uses activated sludge. Municipal wastewater is the majority (∼75%) of the flow (9 to 10 million gallons per day [MGD]) for both plants. The contaminants targeted for removal at both WWTPs are biochemical oxygen demand (BOD), total suspended solids (TSS), ammonium, phosphorus, and pathogenic organisms. Effluent was collected from both plants from the final tank (after chlorination) before discharge into Lake Michigan. Samples were collected in sterile bottles (3 replicates/sample) and placed on ice for transport to the laboratory.

Sediment samples were collected on the same day as wastewater effluent for each of the two locations: 12 sediment samples (sites M1 to M14, 14 July 2015) surrounding the Manitowoc WWTP and five sediment samples (sites S2 to S6, 15 July 2015) surrounding the Sheboygan WWTP. Samples were collected using PONAR, homogenized in 50-ml tubes, aliquoted into cryotubes, and placed in liquid nitrogen for transport to the laboratory. The sampling sites and longitude/latitude coordinates are shown in Fig. 1A and C and Table S1. Three additional sediment samples were collected from the middle of Lake Michigan in August 2015 (LMS11, LMS18, and LMS41) for use as reference sites (Fig. 1E and F).

### DNA extraction and sequencing.

For WWTP effluent samples, 1 liter of each replicate was prefiltered through sterile GF/A (∼1.6-μm-pore-size) filters (Whatman) and then through 0.22-μm-pore-size Sterivex filters (Millipore) using a peristaltic pump. The filters were stored at −80°C until DNA extraction. DNA from the filters was extracted as described by Oh et al. (36). Briefly, the filters were cut into small pieces, mixed well in lysis buffer (50 mM Tris-HCl, 40 mM EDTA, and 0.75 M sucrose), and incubated with 1.15 mg/ml lysozyme and 200 μg/ml RNase at 37°C for 30 min. Samples were subsequently incubated with 1% SDS and 10 mg/ml proteinase K for 2 h with rotation at 55°C. DNA was extracted from the lysate with phenol-chloroform-isoamyl alcohol, precipitated with ethanol, and eluted in Tris-EDTA (TE) buffer. DNA from sediment samples was extracted using the PowerSoil DNA isolation kit (Mo Bio Laboratories, Carlsbad, CA).

DNA from water and sediment samples was sequenced by the DNA Services Laboratory at the University of Illinois at Chicago (IL, USA) using the HiSeq 2500 platform (2 × 100 bp). Total community DNA extracted from WWTP effluent samples collected on the prefilters (1.6 μm representing larger and particle-associated microbes) and three replicates of 0.22-μm-pore-size filters (representing smaller and free-living microbes) were sequenced separately. Rather than analyze attached and free-living communities independently, we combined these data sets into a single whole-community data set.

### Metagenomic sequence processing, assembly, and gene annotation.

The sequencing reads were processed and trimmed as described previously (37). The sequence assemblies of the metagenomes were conducted individually and with WWTP effluent data sets from 1.6-μm prefilters and 0.22-μm filters combined for assemblies, using a range of k-mers (35 to 63) with a hybrid protocol that combines Velvet (38), SOAP*denovo* (39), and Newbler 2.0, as described previously (37). For all samples, Velvet assemblies using two different k-mers (e.g., 35 and 63) were combined with the SOAP assemblies using two different k-mers (e.g., 33 and 61) for the Newbler assembly. The resulting assembled contigs were assigned to protein-coding genes and protein sequences using MetaGeneMark (40).

To determine the coverage of all annotated genes, metagenomic reads were mapped to assembled genes using blastn with default cutoff parameters. The coverage of each gene was computed using the following formula:

(1) $$\text{Gene coverage}=\frac{\text{number of reads mapped to the gene}\;\times \;\text{length of reads}}{\text{length of gene}}$$

### Source tracking analysis.

To look for evidence of genes originating from WWTP effluent in Lake Michigan, we used blastp to align all protein sequences from the lake sediments against those of WWTP effluent. Three samples that were collected from the middle of the lake were also included in this analysis as references. The cutoff threshold used for blastp was: 100% sequence identity of 100% alignment length per query or subject sequence (also called sequence coverage), E value of e^−10^, and bit score of 50 to look for the identical genes in sediments versus WWTPs. The abundance of identical genes from each sediment sample was computed and normalized as described below. The abundances of those genes in sediment samples were plotted using ArcGIS version 10.3.1 using the latitude/longitude coordinates of the corresponding sites.

### Determination and normalization of relative gene abundance.

As the samples (WWTP effluent and lake sediment) used for analysis differ in composition and volume of material processed (see Fig. S1 showing the coverage and diversity of the samples), it was necessary to normalize the gene abundances for comparison. Rather than use the multicopy 16S rRNA gene for normalization, we used the 108 single-copy genes described by Dupont et al. (41). All assembled gene sequences from the metagenomes were compared against a collection of 108 universally conserved single-copy genes using HMMER3 (http://hmmer.janelia.org/), with a default E value threshold of ≤10. The mean value of coverage was then obtained for all genes in metagenome assemblies assigned to these 108 markers. The relative abundance of a gene or a group of genes was normalized using the formula

(2) $$\text{Normalized abundance}=\;\frac{\text{total coverage of a gene or group of genes}}{\text{mean coverage of}\;108\;\text{single-copy marker genes}}$$

### Identification of ARGs, MGE-located ARGs, and virulence factors.

All protein sequences annotated using MetaGeneMark, as described above, in each metagenomic data set were aligned to antibiotic resistance proteins in the Comprehensive Antibiotic Resistance Database (CARD) using blastp, with an E value threshold of e^−10^, 70% sequence similarity cutoff, and bit score of 50 (modified from Hu et al. [42] and Pearson [43]). ARG-like sequences were then classified into different categories (e.g., aminoglycoside, chloramphenicol resistance, etc.) using a Python script to match the blastp result file with ARG category file provided in the database. The coverage of an ARG category was the total coverage of all specific ARGs classified into that category.

To evaluate which ARGs were located on MGEs (“mobile ARGs”), the protein sequences from each metagenome were subjected to a blastp search against the plasmid protein database ACLAME (http://aclame.ulb.ac.be) using the same cutoff threshold described above. The blast result files from the ACLAME plasmid database, which includes ARG-encoding plasmids, were then matched to the blast result files against the CARD for each metagenome. The genes found in both blast results were considered mobile ARGs. The proportion of the total ARGs located on plasmids was calculated using the following formula:

(3) $$\text{Proportion of mobile ARGs in total ARG}\;=\;\frac{\text{total coverage of mobile ARGs}}{\text{total coverage of ARGs}}\times 100$$

In order to track bacterial virulence factors in WWTP effluent, all protein sequences of effluents from both WWTPs were subjected to a blast search against the Virulence Factor Database (44) using a cutoff threshold of 90% sequence identity of 90% alignment length and E value of e^−10^. The identified sequences harboring virulence factors in WWTP effluent were then used with blastp against protein sequences of sediments to look for similar sequences in sediments.

### Taxonomic analysis and detection of ARG- and mobile-ARG-carrying taxa.

The assembled contigs were taxonomically annotated using MyTaxa with a database of 8,942 completed and draft bacterial and archaeal genomes downloaded on 23 March 2016 (45). The composition of each phylum (as a percentage) was calculated as

(4) $$\frac{\text{Total coverage of all genes assigned to a phylum}}{\text{Total coverage of genes assigned to all bacteria}}\times 100$$

The abundance of a taxon (phylum, family, or genus) was normalized using the formula

(5) $$\frac{\text{Total coverage of all genes assigned to a taxon}}{\text{Mean coverage of 108 single-copy marker genes in metagenome}}$$

We used the MyTaxa output to obtain taxonomic information of the assembled genes in order to link gene function (such as ARG/mobile ARGs) to predicted taxonomic origin. A custom script was used to match identified ARGs with taxonomy-assigned genes to identify ARG-carrying taxa or to match MGEs carrying ARGs with taxonomy-assigned genes to identify mobile-ARG-carrying taxa. The proportion of ARG-carrying bacteria of each phylum was then calculated as follows:

(6) $$\frac{\text{Coverage of ARGs belonging to a phylum}}{\text{Total coverage of ARG-carrying bacteria}}\times 100$$

The proportion of the ARG-carrying bacteria or mobile-ARG-carrying bacteria in the total bacterial community was computed as

(7) $$\frac{\text{Total coverage of genes assigned to ARG-carrying bacteria or mobile-ARG-carrying bacteria}}{\text{Total coverage of genes assigned to bacteria}}$$

### Statistical analyses.

The Pearson correlation coefficient between the abundance of certain genes or bacterial taxa found in sediments versus WWTPs with distance from WWTPs was obtained using the R software (https://www.r-project.org). Correlations were deemed significant at a *P* value of <0.05. The relative abundance data of ARGs or bacterial taxa was square root transformed before being used for analysis in order to normalize the different abundances in each data set. No correction for multiple comparisons was performed when analyzing only a select group of taxa.

The distance-decay relationships between the ARG composition or abundance and distance from WWTP outfalls were assessed. The aim of distance-decay analysis is to understand how similarity (or dissimilarity) in a biological community changes in response to distance (46, 47). The ARG profiles of composition (in the presence [1] or absence [0] format) or of abundance of the shared genes that were found in at least one sediment sample and in wastewater effluent were obtained for all samples. Dissimilarity matrices of both ARG composition and abundance in the samples were created based on the Jaccard and Bray-Curtis indices, respectively, using the R vegan package (48). Specifically, we used this approach to evaluate the distance-decay relationship between the distance from the WWTPs and the ARG composition (presence/absence) and abundance. A linear regression model showing the relationship between ARG compositional dissimilarity and the distance from WWTPs was obtained using R. A similar analysis was done to investigate the distance-decay relationship between microbial composition or abundance at the phylum level and distance from WWTP outfall.

### Accession number(s).

All of the raw sequences analyzed in this paper were deposited to the National Center for Biotechnology Information (NCBI) Sequence Read Archive (SRA), with accession number SRP107015.

## Supplementary Material

Supplemental material
