# Supplementary material for: Metagenomics Reveals the Impact of Wastewater Treatment Plants on the Dispersal of Microorganisms and Genes in Aquatic Sediments
Source: Appl Environ Microbiol. 2018 Feb 14;84(5):e02168-17. doi: 10.1128/AEM.02168-17 (PMC5812944; doi:10.1128/AEM.02168-17)
Supplement: Supplemental material [file supp_84_5_e02168-17__index.html]

Supplemental material 

# Metagenomics Reveals the Impact of Wastewater Treatment Plants on the Dispersal of Microorganisms and Genes in Aquatic Sediments

## Supplemental material

- Supplemental file 1 -

  Estimated coverage of metagenomic data sets for Manitowoc (A) and Sheboygan (B) WWTP effluent and Lake Michigan sediment samples (Fig. S1); proportion of genes found in sediment which are identical (100% identity of 100% alignment length) to those from effluents of Manitowoc WWTP (A) and Sheboygan WWTP (B) (Fig. S2); composition of the major bacterial taxa from WWTP effluents and Lake Michigan sediment samples surrounding WWTPs (Fig. S3); taxonomic affiliation at the phylum level of ARG-carrying taxa (Fig. S4); proportions of ARG-carrying bacteria and mobile-ARG-carrying bacteria in Lake Michigan sediments (Fig. S5); latitude and longitude coordinates of the sampling sites (Table S1); supplemental text: digital droplet PCR (ddPCR); example of how to calculate sequencing depth in order to detect one copy of the *sul1* gene from metagenomes of Lake Michigan sediments and WWTP effluents, assuming average genome sizes similar to *E. coli*'s and known cell concentrations in the WWTP effluent and lake sediments (Table S2); single-copy gene *rpoB* in wastewater effluents, quantified by ddPCR (Table S3); metagenomic sequence and assembly statistics (Table S4);

  PDF, 824K
