# Supplementary material for: Metagenomics Reveals the Impact of Wastewater Treatment Plants on the Dispersal of Microorganisms and Genes in Aquatic Sediments
Source: Appl Environ Microbiol. 2018 Feb 14;84(5):e02168-17. doi: 10.1128/AEM.02168-17 (PMC5812944; doi:10.1128/AEM.02168-17)
Supplement: Supplemental material [file AEM.02168-17_zam005188357s1.pdf]

Metagenomic analysis reveals the impact of wastewater treatment plants on the dispersal of microorganisms and genes in aquatic sediments

Supporting Information

Chu T.T. Binh[1,2,3], Morgan L. Petrovich[2], Adit Chaudhary[1], Dorothy Wright[1], Brian Murphy[3], George Wells[2], Rachel Poretsky[1*]

[1] Department of Biological Sciences, University of Illinois at Chicago, Chicago, IL, USA.
[2] Department of Civil and Environmental Engineering, Northwestern University, Evanston, IL, USA.
[3] Dept. of Medicinal Chemistry & Pharmacognosy, University of Illinois at Chicago, Chicago, IL, USA

*Corresponding author: Rachel Poretsky
Email: microbe@uic.edu

The supporting information section consists of 7 pages and includes 3 tables and 5 figures.

(A)

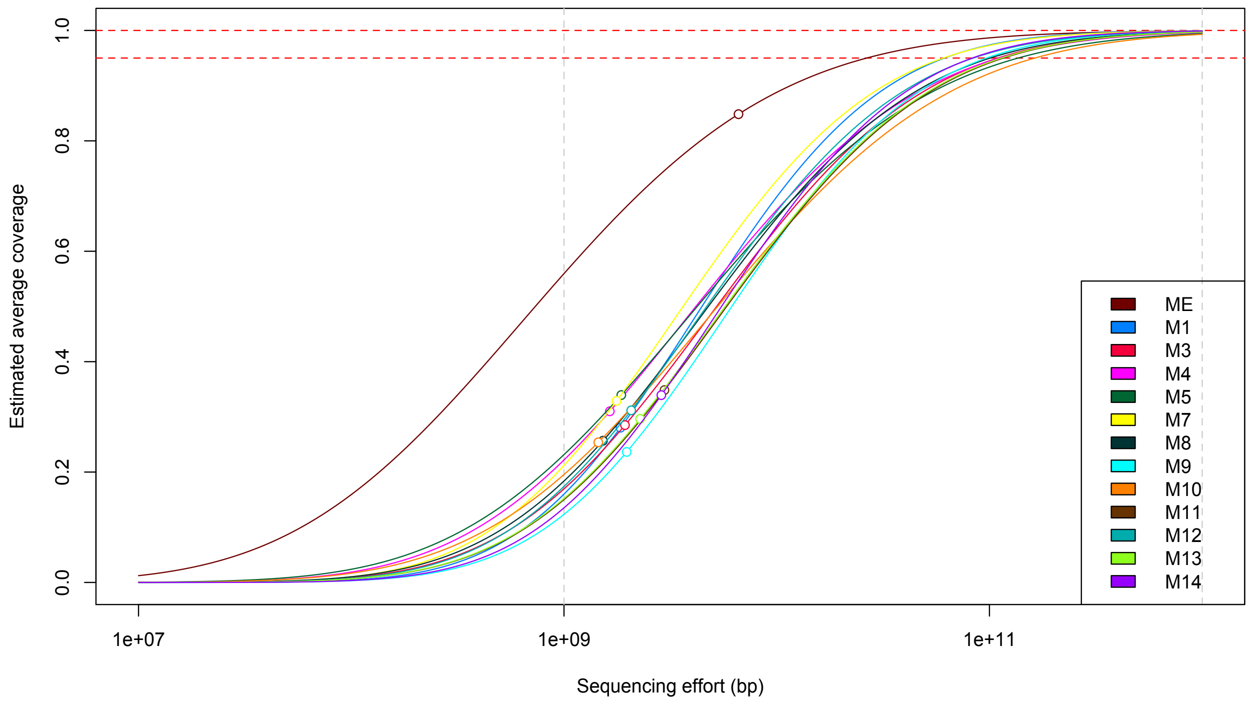

(B)

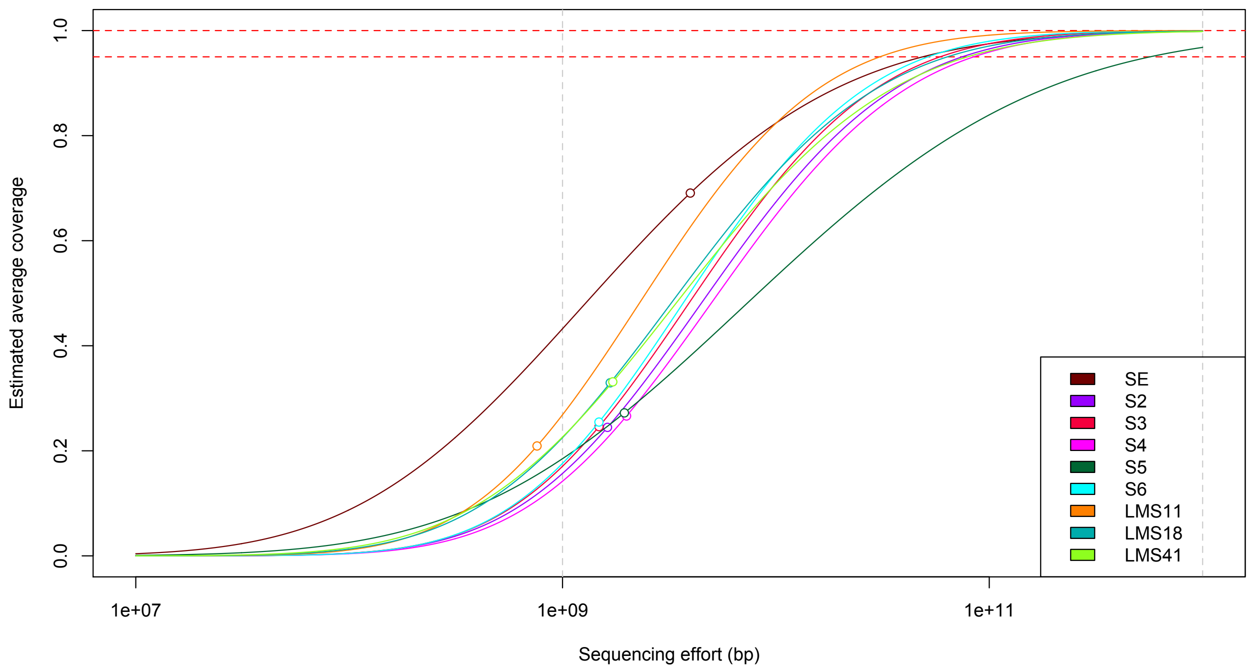

Fig S1. Estimated coverage of metagenomic datasets for Manitowoc (A) and Sheboygan (B) WWTP effluent and Lake Michigan sediment samples. ME, SE: effluent from Manitowoc and Sheboygan WWTP, respectively. M1-M14: sites near Manitowoc WWTP effluent outfall; S2-S6: sites near Sheboygan WWTP effluent outfall; LMS11, 18, 41: reference sites in the middle of the Lake Michigan. Nonpareil curves were constructed using the protocol described by (1).

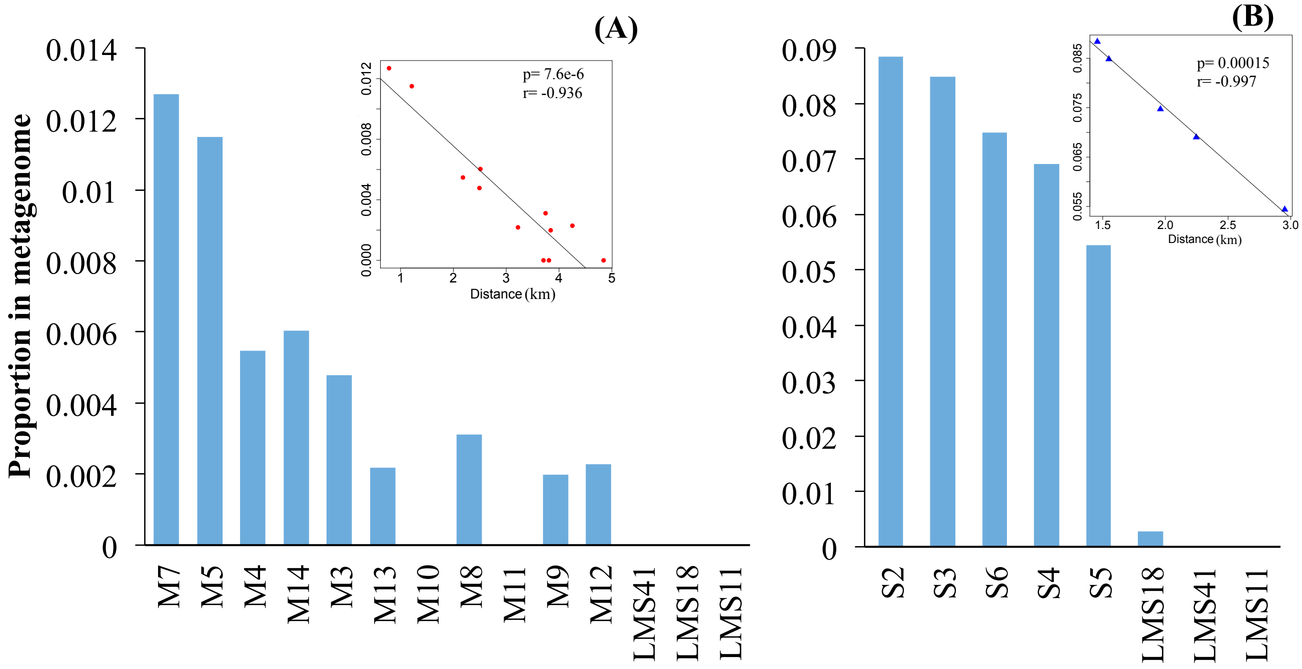

Fig S2. Proportion of the genes found in sediment which are identical (100% identity of 100% alignment length) to those from effluents of Manitowoc WWTP (A) and Sheboygan WWTP (B). The insets showed the negative correlation between proportion of identical genes and distance from effluent outfalls. Three samples collected in the middle of the Lake Michigan were used as references. Proportion = number of different genes found in sediment identical to those in WWTP effluent /total number of the genes in metagenomes *100. Order of the sites in figure, from left to right, corresponds to increasing distance from WWTPs.

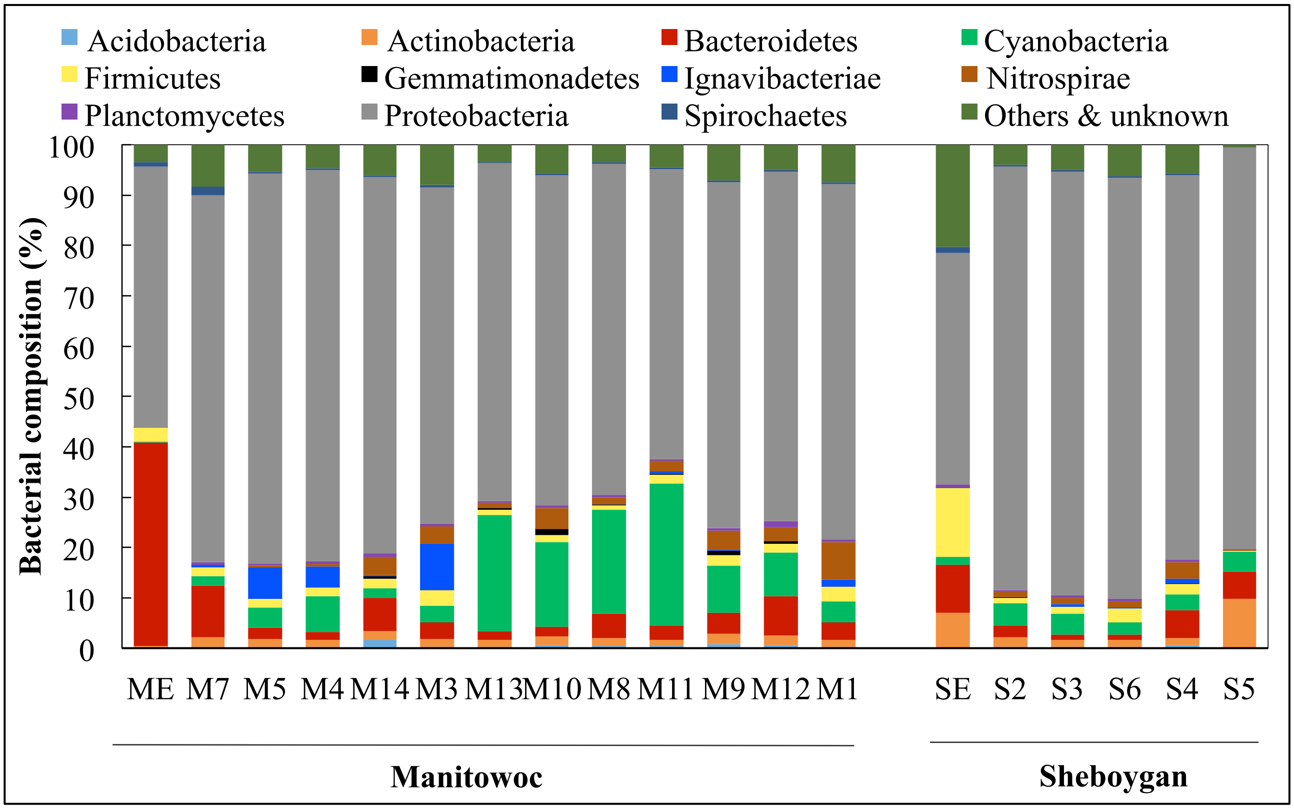

Fig S3. Composition of the major bacterial taxa from WWTP effluents and Lake Michigan sediment samples surrounding WWTPs. ME, SE: effluent from Manitowoc and Sheboygan WWTP, respectively. M1-M14: sites around Manitowoc WWTP, S2-S6: sites around Sheboygan WWTP. Only the phyla which are > 1% in at least one sample are shown. Order of the sites in figure, from left to right, corresponds to increasing distance from WWTPs.

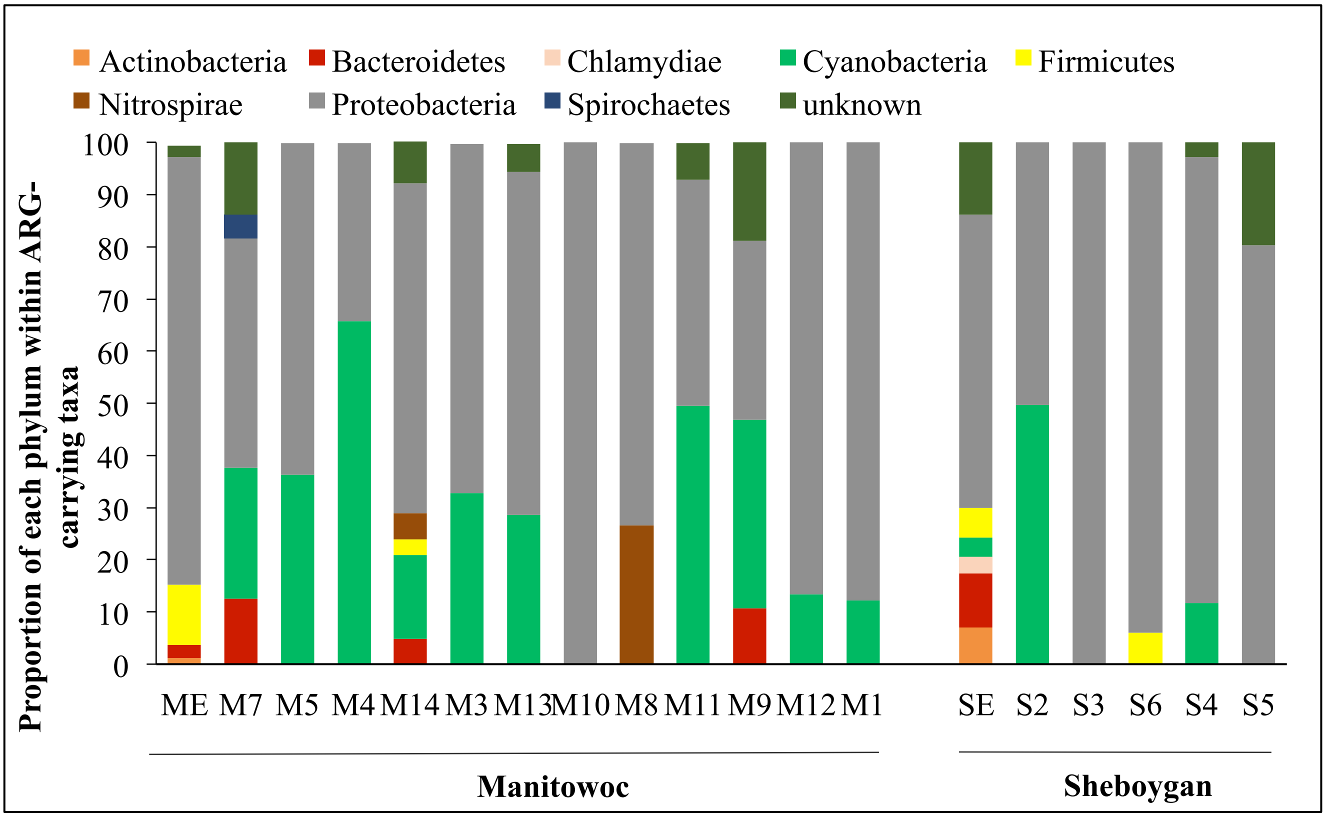

Fig S4. Taxonomic affiliation at the phylum level of ARG-carrying taxa. ME, SE: effluent from Manitowoc and Sheboygan WWTP, respectively; M1-M14: sites around Manitowoc WWTP; S2-S6: sites around Sheboygan WWTP. Only phyla which are > 1% in at least one sample are shown. Order of the sites in figure, from left to right, corresponds to increasing distance from WWTPs.

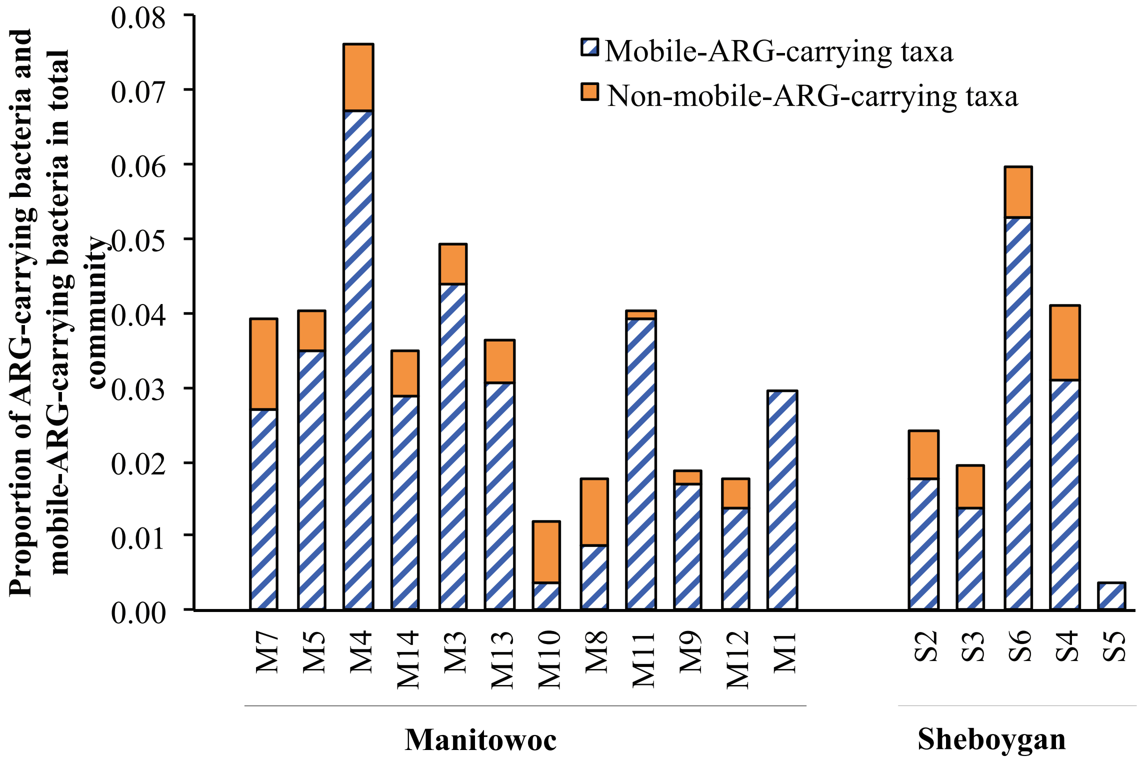

Fig S5. Proportion of ARG-carrying bacteria and mobile-ARG-carrying bacteria in Lake Michigan sediments (M1-M14, S1-S6).

Order of the sites in figure, from left to right, corresponds to increasing distance from WWTPs.

Table S1. Latitude and longitude coordinates of the sampling sites

Site	Latitude	Longitude	Site	Latitude	Longitude
M1	44º 06.263 N	087º 36.210 W	S2	43º 43.7814 N	87º 41.9026 W
M3	44º 05.907 N	087º 38.047 W	S3	43º 43.7651 N	87º 41.7583 W
M4	44º 05.709 N	087º 38.416 W	S4	43º 43.7764 N	87º 41.0879 W
M5	44º 05.315 N	087º 38.733 W	S5	43º 44.1410 N	87º 40.8049 W
M6	44º 05.133 N	087º 38.880 W	S6	43º 43.4339 N	87º 41.1024 W
M7	44º 04.736 N	087º 38.831 W	Sheboygan WWTP	43.718756	-87.708365
M8	44º 04.402 N	087º 36.635 W			
M9	44º 04.231 N	087º 36.635 W			
M10	44º 04.852 N	087º 36.566 W	LMS11	42°23.0052 N	087°00.0180 W
M11	44º 05.308 N	087º 36.532 W	LMS18	42°43.9751 N	087°00.1094 W
M12	44º 05.795 N	087º 36.383 W	LMS41	44°44.2031 N	086°43.2907 W
M13	44º 05.468 N	087º 37.041 W			
M14	44º 05.129 N	087º 37.479 W			
Manitowoc WWTP	44.0823076	-87.6579573			

Digital droplet PCR (ddPCR):

A ddPCR assay was used to quantify a single-copy marker gene (*rpo*B) (2) and sulfonamide resistance gene *sul1* (3). ddPCR was performed using QX200 ddPCR system (Bio-Rad, Pleasanton, CA, USA) with the ddPCR EvaGreen Supermix (Bio-Rad). The total 20 μL reaction mixtures consisted of 10 μL of PCR premix, primers (final concentration 20 μM), template DNA. The entire 20 μL reaction mixture was loaded into a sample well of the disposable droplet generator cartridge (Bio-Rad) with 70 μL of QX200 Droplet Generation Oil for EvaGreen (Bio-Rad) and placed in the QX200 droplet generator (Bio-Rad) which uses specially developed reagents and microfluidics to partition each sample into 20,000 nanoliter-sized droplets. After processing, the droplets were transferred to a 96-well PCR plate. The plate was heat-sealed with pierceable foil. PCR amplification was carried out with temperature cycles: enzyme activation 95°C-5min, denaturing 95°C-30s, annealing - 1min (depending on primers used), stabilization 12°C.

The amplification of the genes were detected and quantified using QX200 droplet reader, which counts the fluorescent positive and negative droplets to calculate target DNA concentration with QuantaSoft software (Bio-Rad, CA, USA). All samples were tested in three replicates.

Table S2. Example of how to calculate sequencing depth in order to detect one copy of *sul1* genes from metagenomes of Lake Michigan sediments and WWTP effluents, assuming average genome sizes similar to *E.coli* and known cell concentrations in the WWTP effluent and lake sediments.

Sample	Type	# of genes/g or /L	*sul1* (copies/g or/L)	# of gene need to sequence to find 1 copy of *sul1*	sequencing depth need (GB)
ME-1	effluent	3E+12	1070125	2.8E+06	4
ME-2	effluent	3E+12	947625	3.2E+06	4
ME-3	effluent	3E+12	895125	3.4E+06	4
M1-1	sediment	3E+11	84	3.6E+09	4762
M1-2	sediment	3E+11	260	1.2E+09	1538
M1-3	sediment	3E+11	64	4.7E+09	6250
M5-1	sediment	3E+11	1500	2.0E+08	267
M5-2	sediment	3E+11	620	4.8E+08	645
M5-3	sediment	3E+11	420	7.1E+08	952
M7-1	sediment	3E+11	180	1.7E+09	2222
M7-2	sediment	3E+11	76	3.9E+09	5263
M7-3	sediment	3E+11	116	2.6E+09	3448
SE-1	effluent	3E+12	180250	1.7E+07	22
SE-2	effluent	3E+12	234150	1.3E+07	17
SE-3	effluent	3E+12	414050	7.2E+06	10
S1-1	sediment	3E+11	80	3.8E+09	5000
S1-2	sediment	3E+11	32	9.4E+09	12500
S1-3	sediment	3E+11	40	7.5E+09	10000
S4-1	sediment	3E+11	156	1.9E+09	2564
S4-2	sediment	3E+11	116	2.6E+09	3448
S4-3	sediment	3E+11	88	3.4E+09	4545
S5-1	sediment	3E+11	60	5.0E+09	6667
S5-2	sediment	3E+11	580	5.2E+08	690
S5-3	sediment	3E+11	160	1.9E+09	2500

Table S3. Single-copy gene *rpo*B in wastewater effluents quantified by ddPCR

**Sample**	***rpo*B (copies/L)**
ME-1 (Manitowoc WWTP effluent, replicate 1)	3.60E+06
ME-2 (Manitowoc WWTP effluent, replicate 2)	2.70E+06
ME-3 (Manitowoc WWTP effluent, replicate 3)	3.61E+06
SE-1 (Sheboygan WWTP effluent, replicate 1)	9.81E+05
SE-2 (Sheboygan WWTP effluent, replicate 2)	1.37E+06
SE-3 (Sheboygan WWTP effluent, replicate 3)	1.35E+06

Table S4. Metagenomic sequence and assembly statistics

Sample	Sample location	Number of reads	Number of contigs	Number of genes	Proportion of identical gene *vs.* Manitowoc WWTP effluent (%)	Proportion of identical gene *vs.* Sheboygan WWTP effluent (%)	Proportion of genes classified to genus level (%)
ME	Manitowoc watewater treatment plant (WWTP) effluent	66,731,046 *	112,065	284,201			50.13
M1	Lake Michigan sediment, site 1 near Manitowoc WWTP	19,747,137	21,453	35,161	0		44.58
M3	Lake Michigan sediment, site 3 near Manitowoc WWTP	20,633,604	25,117	41,874	0.00478		46.83
M4	Lake Michigan sediment, site 4 near Manitowoc WWTP	17,648,342	21,669	36,546	0.00547		43.51
M5	Lake Michigan sediment, site 5 near Manitowoc WWTP	19,920,426	26,330	43,473	0.01150		45.51
M7	Lake Michigan sediment, site 7 near Manitowoc WWTP	18,892,935	29,975	55,166	0.01269		43.15
M8	Lake Michigan sediment, site 8 near Manitowoc WWTP	16,272,551	20,867	32,127	0.00311		40.39
M9	Lake Michigan sediment, site 9 near Manitowoc WWTP	21,218,375	34,174	50,337	0.00199		44.47
M10	Lake Michigan sediment, site 10 near Manitowoc WWTP	15,580,432	27,718	44,398	0		41.76
M11	Lake Michigan sediment, site 11 near Manitowoc WWTP	31,803,102	45,567	80,602	0		39.63
M12	Lake Michigan sediment, site 12 near Manitowoc WWTP	22,124,964	53,163	87,392	0.00229		41.55
M13	Lake Michigan sediment, site 13 near Manitowoc WWTP	24,444,408	28,733	45,911	0.00218		39.64
M14	Lake Michigan sediment, site 14 near Manitowoc WWTP	30,799,471	60,177	99,439	0.00603		43.54
SE	Sheboygan WWTP effluent	40,043,864 *	79,944	224,534			44.51
S2	Lake Michigan sediment, site 2 near Sheboygan WWTP	16,595,569	11,695	13,033		0.08836	42.80
S3	Lake Michigan sediment, site 3 near Sheboygan WWTP	18,035,433	8,122	18,451		0.08482	41.67
S4	Lake Michigan sediment, site 4 near Sheboygan WWTP	22,454,246	24,793	41,338		0.06901	42.48
S5	Lake Michigan sediment, site 5 near Sheboygan WWTP	22,255,105	14,007	22,980		0.05438	41.74
S6	Lake Michigan sediment, site 6 near Sheboygan WWTP	16,556,166	13,225	22,072		0.07470	41.82
LMS11	Lake Michigan sediment, site 11, in the middle of the lake	8,474,557	6,226	11,482	0	0	68.25
LMS18	Lake Michigan sediment, site 18, in the middle of the lake	18,476,347	17,752	35,406	0	0.00282	57.04
LMS41	Lake Michigan sediment, site 41, in the middle of the lake	19,072,732	17,385	34,158	0	0	58.08

* combined from metagenomes of pre-filter (1.6μm) and 3 replicates of 0.22μm filters

References:

1. Rodriguez-R LM, Konstantinidis KT. 2014. Nonpareil: A redundancy-based approach to assess the level of coverage in metagenomic datasets. Bioinformatics 30:629-635.

2. Case RJ, Boucher Y, Dahllof I, Holmstrom C, Doolittle WF, and Kjelleberg, S. 2007. Use of 16S rRNA and rpoB genes as molecular markers for microbial ecology studies. Appl Environ Microbiol 73:278-288.

3. Heuer H, Smalla K. 2007. Manure and sulfadiazine synergistically increased bacterial antibiotic resistance in soil over at least two months. Environ Microbiol 9:657-666.
